# Supplementary material for: Does an Extraoral Suction Device Reduce Aerosol Generation and Prevent Droplet Exposure to the Examiner during Esophagogastroduodenoscopy?
Source: J Clin Med. 2023 Mar 29;12(7):2574. doi: 10.3390/jcm12072574 (PMC10094833; doi:10.3390/jcm12072574)
Supplement: Supplementary file 1 [file jcm-12-02574-s001.zip › jcm-2278070-supplementary.pdf]

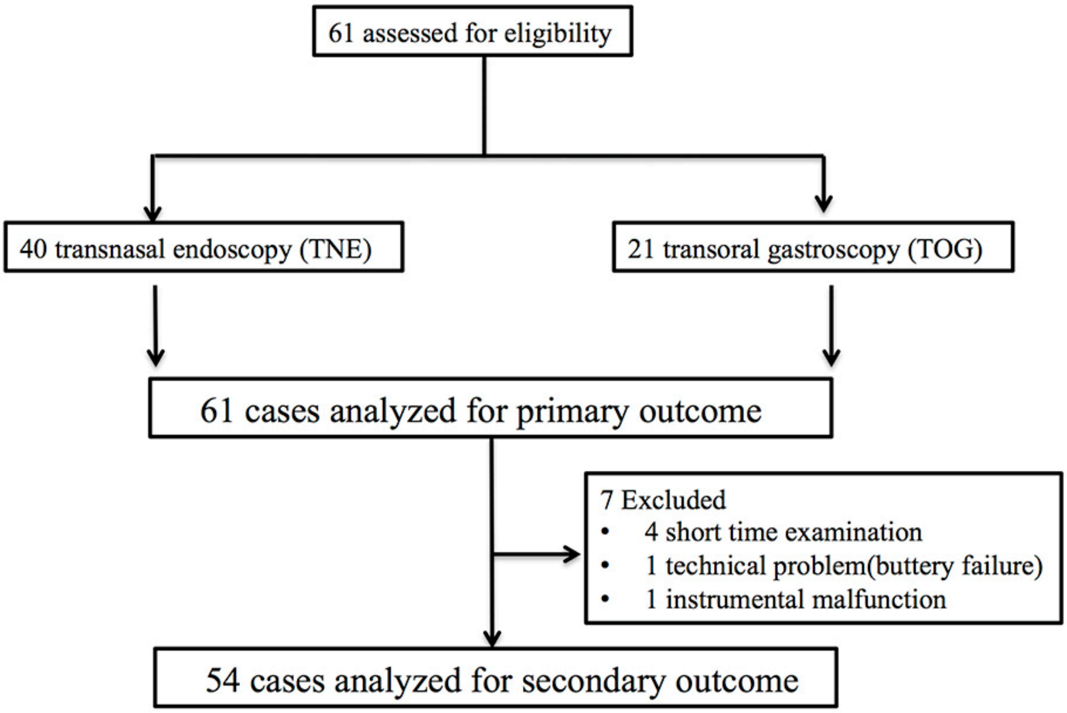

Supplemental Figure S1. Patient enrollment. TNE, transnasal endoscopy. TOG, transoral gastroscopy

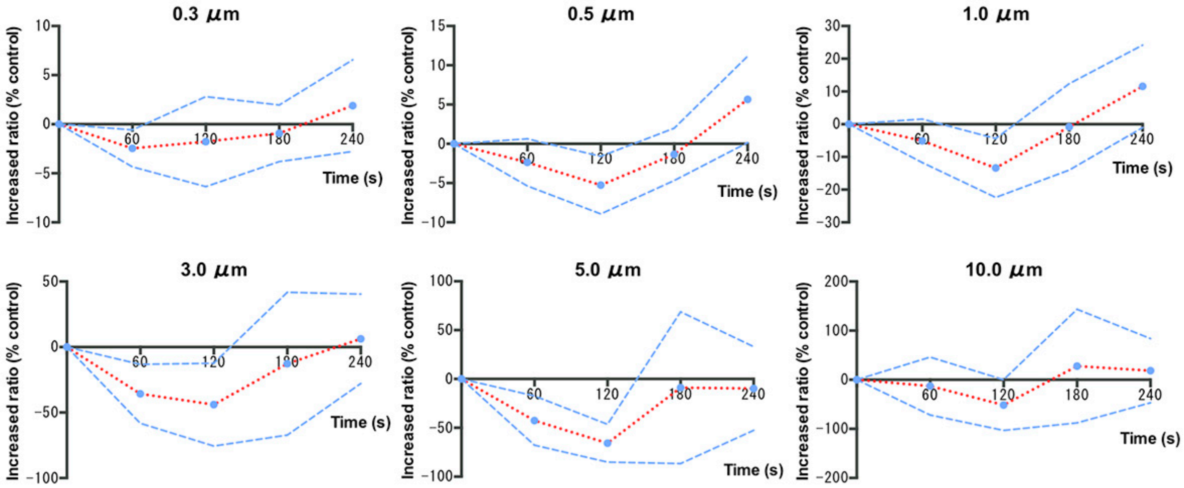

Supplemental Figure S2. Percentage increase in aerosol particles of 0.3, 0.5, 1, 3, 5, and 10 μm in size at 60, 120, 180 and 240 seconds from the start of esophagogastroduodenoscopy without an extraoral suction device (n = 8). The lines represent the mean particle count of the eight patients. The blue dotted line indicates the 95% confidence interval.

Supplemental Table S1. Comparison of baseline characteristics of nasal endoscopy (TNE) and transoral endoscopes (TOE) with extraoral suction device.

	Transnasal endoscopy<br>n=40	Transoral endoscopy<br>n=21	P-value
**Mean age ± SD, y**	61.9±15.4	71.0±16.2	0.0197
**Male sex, no (%)**	22 (61.6)	14 (66.7)	0.4234
**Medical history, n(%)**	15 (37.5)	6 (28.0)	0.5773
**Mean procedure time ± SD, secs**	203.1± 55.7	160.3± 66.4	0.0013
**Cough, n(%)**	11(27.5)	6(28.6)	1.0000
**Reflex vomitting, n(%)**	6(15.0)	6(28.6)	0.3093
**Sneezing, n(%)**	0 (0)	0 (0)	
**Burping, n(%)**	8(20)	11(52.4)	0.0184
**Body movement, n(%)**	1(2.5)	2(9.5)	0.2704
**Interventions, n(%)**			
**No**	33(82.5%)	20(95.2%)	0.2434
**Biopsy**	8 (13.1)	8 (13.1)	

Supplemental Table S2. Comparison of changes in aerosol counts and ATP levels between transnasal endoscopy (TNE) and transoral endoscopy (TOE) with an extraoral suction device.

	**Transnasal endoscopy**			**Transoral endoscopy**			
	Before	After	Δ(After-Before)	Before	After	Δ(After-Before)	P-value
Mean counts ($x10^6/m^3$) ± SD							
0.3 μm	25.6 ± 13.2	28.8 ± 15.5	3.2 ± 3.0	26.0 ± 16.2	29.4 ± 19.3	28.9 ± 16.1	0.5641
0.5 μm	2.5 ± 1.5	2.8 ± 1.8	0.3 ± 0.4	2.8 ± 2.2	3.1 ± 2.7	2.9 ± 2.1	0.8913
1.0 μm	0.2 ± 0.8	0.3 ± 0.1	0.01 ± 0.06	0.3 ± 1.3	2.6 ± 1.5	0.3 ± 1.2	0.7905
Mean counts ($x10^3/m^3$) ± SD							
3.0 μm	12.4 ± 11.4	9.5 ± 5.2	-2.9 ± 10.4	9.4 ± 3.6	7.8 ± 3.7	-1.7 ± 5.5	0.6818
5.0 μm	3.7 ± 4.8	2.8 ± 2.1	-0.9 ± 4.5	2.4 ± 1.5	1.9 ± 1.0	-4.5 ± 1.8	0.6703
10.0 μm	2.6 ± 3.5	1.7 ± 1.5	-0.9 ± 3.3	1.5 ± 1.0	1.2 ± 0.6	-3.2 ± 1.1	0.3165
ATP level (RLU)	8.4 ± 7.6	83.9 ± 275.9	75.5 ± 277.3	8.0 ± 7.6	26.4 ± 36.4	18.4 ± 36.6	0.0974
